# Time‐related differences and influencing factors of acute stress symptoms in university students exposed to suicide

**DOI:** 10.1002/pcn5.70323

**Published:** 2026-03-25

**Authors:** Isamu Yamazaki, Tohru Takahashi, Daimei Sasayama, Hiroshi Morita, Shinsuke Washizuka

**Affiliations:** ^1^ Center for Health, Safety and Well‐being, Shinshu University Matsumoto Nagano Japan; ^2^ Department of Psychiatry Shinshu University School of Medicine Matsumoto Nagano Japan; ^3^ Department of Child and Adolescent Developmental Psychiatry Shinshu University School of Medicine Matsumoto Nagano Japan

**Keywords:** acute stress disorder, post‐traumatic stress disorder, students, suicide, university

## Abstract

**Aim:**

Although clarifying the immediate psychological impact on students exposed to suicide is essential, standardized suicide postvention protocols in university settings are currently lacking. This study examined the temporal differences and contributing factors in the psychological impact of suicide exposure among university students using postvention records from a single Japanese university.

**Methods:**

We collected and analyzed the records of 32 student suicide cases at Shinshu University between 2009 and 2023. Of these, 10 were selected for further analysis based on the availability of both postvention interview records and scores from the Japanese‐language version of the Impact of Event Scale‐Revised (IES‐R‐J). Among the 136 students who received postvention support, data from 129 students exposed to suicide were included in the analysis. Time‐related differences in the initial IES‐R‐J scores were analyzed using analysis of variance, and the factors influencing the scores were examined using multiple regression analysis.

**Results:**

Postvention was implemented in 17 of 32 suicide cases. In the 10 selected cases, higher IES‐R‐J scores were associated with being female, discovering or witnessing the suicide, fewer days elapsed since the suicide, a documented relationship with the deceased, and personal vulnerability noted in records.

**Conclusion:**

This study identified key factors associated with acute stress responses among university students exposed to suicide. Although symptoms generally decline over time, several factors were associated with increased severity. Suicide prevention in universities should prioritize support for individuals at greater risk based on the risk factors identified in this study.

## Graphical abstract

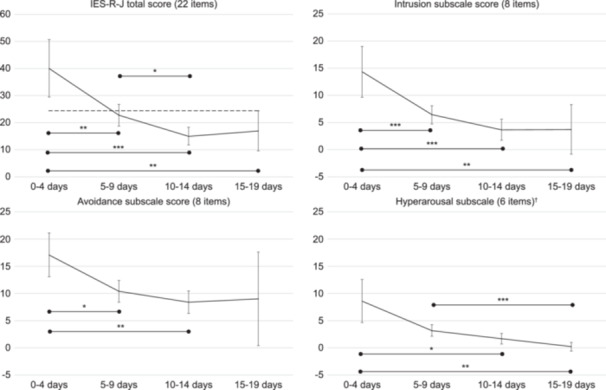

## INTRODUCTION

Japan has the highest suicide rate among the Group of Seven (G7) countries.^1^ Suicide is therefore recognized as a major public health and social concern in Japan. The suicide rate among young people has continued to increase,^1^ making it the leading cause of death among individuals aged 15–24 years,^2^ the typical age group of university students. It has been the leading cause of death among Japanese university students since 1996. Although the rate showed a downward trend, it rose again in 2020, coinciding with the onset of the COVID‐19 pandemic.^3^

Suicide can have a profound psychological impact on those exposed to it.^4^, ^5^, ^6^, ^7^, ^8^, ^9^, ^10^, ^11^, ^12^ Exposure to suicide (i.e., knowing an individual who died by suicide) has been reported to increase the risk of suicidal behaviors.^4^, ^5^ This impact may be particularly strong during adolescence and young adulthood compared with other life stages.^6^, ^7^ Suicide exposure increases the risk of developing post‐traumatic stress disorder (PTSD).^8^, ^9^, ^10^, ^11^ Reported risk factors for PTSD following exposure to suicide include sex, age, prior psychiatric history, degree of exposure, social network proximity, relationship with the deceased, family environment, and stressful life events.^10^ However, most previous studies have primarily examined medium‐ to long‐term psychological outcomes, such as PTSD, depression, and increased suicidal behavior.^6^, ^10^, ^11^, ^12^ Relatively few studies have focused on acute stress symptoms that arise immediately following suicide exposure or on the factors contributing to these reactions.^13^

To minimize the psychological burden of suicide exposure, postvention interventions are often implemented. Shneidman describes the aim of postvention as follows: “Its purpose is to help survivors live longer, more productively, and less stressfully than they would otherwise.”^14^ Suicide postvention involves targeted interventions designed to reduce psychological distress among affected individuals exposed to suicide.^15^ Common strategies include individual counseling and group‑based psychoeducation for individuals who discovered or were close to the deceased, aimed at supporting their mental well‑being and reducing the risk of suicide contagion.^15^

According to Brock et al., psychological support in response to school‐related crises, including suicide, should be prioritized based on three factors: the level of exposure to a crisis event, familiarity with a crisis victim, and individual vulnerabilities.^16^ Despite these frameworks, evidence on suicide postvention in university settings remains limited, and standardized protocols are yet to be established.^17^ To develop effective postvention strategies, examining how PTSD risk factors, the three prioritization criteria proposed by Brock et al., and the passage of time since suicide influence the identification of appropriate postvention targets is necessary.

At Shinshu University, psychologists and psychiatrists provide mental health support^18^ and suicide prevention for students.^19^ When a suicide occurs, the university conducts a factual investigation,^20^ shares information with relevant parties, provides an immediate crisis response, and implements postvention measures as needed.^21^ Postvention records are maintained at the faculty level by psychologists and psychiatrists, reflecting documentation of interventions and organizational procedures. Building on these institutional practices, longitudinal investigations of student suicides^18^, ^20^ and reviews of postvention records and intervention procedures have been conducted.^15^, ^19^

However, to the best of our knowledge, time‐related changes in acute stress reactions among students exposed to suicide, and the factors influencing symptom severity, have not been systematically examined. To address this gap, we analyzed postvention records collected at Shinshu University. We hypothesized that variables such as time elapsed since suicide, sex (a known PTSD risk factor), relationship with the deceased, and pre‐existing mental health vulnerabilities would be associated with scores on the Japanese‐language version of the Impact of Event Scale‐Revised (IES‐R‐J),^22^ a self‐report questionnaire used to measure post‐traumatic stress symptoms.

## METHODS

### Analysis of suicide cases and postvention

**Figure 1:**
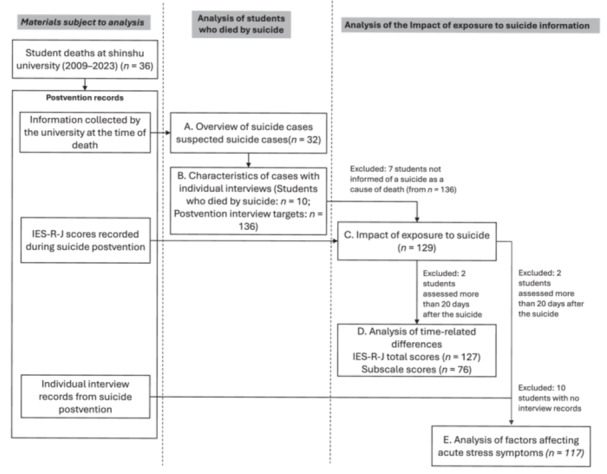
Flowchart of the current study. This flowchart illustrates the process of selecting participants and the materials analyzed. Participants were selected from university students exposed to suicide, and the analyzed materials were postvention records. IES‐R‐J, Japanese‐language version of the Impact of Event Scale‐Revised.

Student suicides at Shinshu University between 2009 and 2023, the only period with available data, were investigated. Among 36 reported student deaths (including those with unknown causes), 32 suspected suicides were confirmed and included in the analysis. The overall procedure is illustrated in Figure 1.

#### Overview of suicide cases

For each of the 32 suspected suicide cases, university‐collected materials were examined to extract the following details: sex of the deceased, method of suicide, duration from university enrollment to suicide, history of using counseling services, living arrangements, whether the student was discovered or witnessed at the scene, whether postvention was conducted, and whether the cause of death was disclosed to the students.

#### Characteristics of cases with individual interviews

From the 32 suspected suicide cases, 10 cases were selected that included both individual postvention interview records and IES‐R‐J^22^ scores. Data from 136 students exposed to these 10 cases and who received individual postventions were analyzed. Postvention recipients were identified among students in the same seminar, laboratory, or extracurricular clubs as the deceased, or those deemed by faculty/staff to have had close relationships with the deceased. These determinations used university‐collected information, in consultation with supervising faculty members and specialists at the Center for Health, Safety and Well‐Being.

#### Impact of exposure to suicide

Among the 136 students who received postvention, 129 were informed that the cause of death was suicide (including two international students). International students received verbal explanations during IES‐R‐J administration to ensure comprehension. “Exposure to suicide” was defined as confirmed knowledge of the deceased student's suicide (e.g., via direct witnessing, student networks, or university disclosure). Disclosure occurred if students were likely aware or affected; nondisclosure was applied per family requests or minimal contact. Decisions were made by specialists using university data and faculty input. Non‐exposed students (uninformed of suicide) were therefore excluded from the analysis.

Students were categorized by IES‐R‐J cutoff (<25 vs. ≥25): cross‐tabulations examined sex, enrollment year, faculty, interview selection reason, and discovery/witness status. Cells with ≤5 cases used Fisher's exact or Fisher–Freeman–Halton tests.

#### Analysis of time‐related differences

One‐way analysis of variance (anova) assessed time‐related differences in acute stress symptoms with total IES‐R‐J score as the dependent variable and time since exposure as the independent variable. Because the exact date of exposure was difficult to determine, we used the number of days from suicide to the administration of the IES‐R‐J as a proxy, grouping the data into 5‐day intervals. Two students assessed 20 days or more after the suicide were excluded because of small sample sizes, leaving 127 students assessed within the 0–19‐day period (grouped as 0–4, 5–9, 10–14, and 15–19 days).

For the subset of 76 students with available IES‐R‐J subscale scores, we conducted anovas for each subscale to analyze the effect of elapsed time and Tukey's honestly significant difference (HSD) test for multiple comparisons between groups. Levene's test was used to assess the homogeneity of variance across groups. When homogeneity was not confirmed, Welch's anova (robust test of equality of means) and the Games–Howell method were used for multiple comparisons.

To assess bias arising from systematic differences between participants with and without complete subscale data in the time‐related pattern observed in the full‐sample analysis of total IES‐R‐J scores (*n* = 127), we conducted a sensitivity analysis using the same subset of participants (*n* = 76) for whom all subscale scores were available.

#### Analysis of factors affecting acute stress symptoms

Stepwise multiple regression analysis using the IES‐R‐J total score as the dependent variable was done to explore the factors associated with acute stress symptoms. The following independent variables were included: sex (0 = male, 1 = female), being a discoverer or witness (0 = no, 1 = yes), being in the same academic year as the deceased (0 = no, 1 = yes), number of days from suicide to the IES‐R‐J assessment, presence of a description of the student's relationship with the deceased in the interview record (0 = absent, 1 = present), and presence of vulnerability‐related information in the interview record (0 = absent, 1 = present). Dummy variables were applied to the categorical data.

### Psychological scale

The 22‐item IES‐R‐J^22^, ^23^ was used to assess post‐traumatic stress via three subscales: intrusion (8 items), avoidance (8 items), and hyperarousal (6 items). Each item was rated on a 5‐point Likert scale, where 0 = “not at all,” 1 = “a little,” 2 = “moderately,” 3 = “quite a bit,” and 4 = “extremely.” A cutoff score of 24/25 is the optimal threshold for identifying probable PTSD.^22^ We analyzed only the initial score for the participants who completed the IES‐R‐J multiple times.

### Statistical analysis

All statistical analyses were performed using IBM spss Statistics for Windows, version 29. The significance level was set at 5%.

### Ethical considerations

This study was approved by the Biological and Medical Research Ethics Committee of Shinshu University School of Medicine (Approval No. 6144). This was a retrospective study based on records, using only non‐identifiable personal information. Details of the study are publicly available on the university's website, and participants were able to opt out of the study (https://www.shinshu-u.ac.jp/institution/health/docs/mental_research2024.pdf). The authors declare no conflicts of interest directly relevant to the content of this article.

The following safety measures were implemented in this study. Psychological debriefing (potentially harmful)^24^ was avoided. The IES‐R‐J was administered as a concurrent assessment during the initial individual interviews. Specialist follow‐up was provided for students with high IES‐R‐J scores or those requesting ongoing support. Other participants were monitored as needed by their supervisors. Follow‐up interviews were led by psychologists employed at Shinshu University (holding Clinical Psychologist certification from the Japanese Certification Board for Clinical Psychologists and/or national Public Psychologist license) or psychiatrists when required. Although no specific stopping rules were predefined, any participant exhibiting signs of adverse reactions during the postvention procedures would be referred to a psychiatrist. External counseling referrals were offered as needed.

## RESULTS

### Overview of suicide cases

Table 1 summarizes characteristics of the 32 suicides at Shinshu University between 2009 and 2023. Of the 32 suicides, 30 were male (94%), and 2 were female (6%). The most common suicide method was hanging (20 cases, 63%). The highest incidence was observed among fourth‐year (senior‐year) undergraduate students (10 cases, 31%). In 25 cases (78%), there was no prior use of university counseling services. While approximately 70% of students at Shinshu University lived alone,^20^ the rate was higher among those who died by suicide (30 cases, 94%, *p* < 0.005).

**Table 1:** Characteristics of all suicide cases (*n* = 32).

Characteristics of suicide cases	Number of cases	
Sex		
Male	30	(94%)
Female	2	(6%)
Cause of death		
Hanging	20	(63%)
Gas	3	(9%)
Jumping	3	(9%)
Other	6	(19%)
Years from university enrollment to suicide		
Undergraduate program	26	(81%)
1–2 years	2	(6%)
3 years	8	(25%)
4 years	10	(31%)
5 or more years	6	(19%)
Graduate program	6	(19%)
1 year	3	(9%)
2 years	3	(9%)
History of using counseling services		
Yes	7	(22%)
No	25	(78%)
Living situation		
Living alone	30	(94%)
Living with family	2	(6%)
Discoverer/witness (overlap present)		
Student/friend	10	(31%)
Civilian	5	(16%)
Family	4	(13%)
Faculty members	3	(9%)
Residents and building staff	2	(6%)
Police	1	(3%)
Unknown	9	(28%)
Postvention intervention (record availability)		
Individual interview only	10	(31%)
Group psychoeducation only	2	(6%)
Both implemented	5	(16%)
No record	15	(47%)
Cause of death communicated to related students (overlap present)		
Suicide	12	(38%)
Non‐suicide	7	(22%)
Unknown/not communicated	14	(44%)

University‐affiliated discoverers or witnesses were identified in 12 cases (38%). Among these, 10 involved students or friends, and 3 involved faculty members, with one case involving both groups. Postventions—individual interviews, group psychoeducation, or both—were conducted in 17 cases (53%). Of these 17 cases, the cause of death was explicitly communicated to students as suicide in 12 cases (38%). For the remaining 15 cases (47%), no postvention was conducted, or records were unavailable. Reasons for non‐implementation of postvention in these 15 cases included family requests for nondisclosure, lack of close student contacts, and the low likelihood of student awareness.^15^

### Characteristics of cases with individual interviews

Table 2 shows the characteristics of the ten completed suicide cases for which both individual interview records and IES‐R‐J scores were available. The characteristics observed in these cases were generally similar to those of the overall suicide cases shown in Table 1. The majority were male (90%), hanging was the most frequent method (90%), many were fourth‐year (senior‐year) undergraduates (40%), few had utilized counseling services (10%), and a high proportion lived alone (80%). University‐affiliated individuals, particularly students and friends (six cases), were common discoverers or witnesses. Notably, in these 10 cases, the disclosed cause of death was most frequently identified as suicide (90%).

**Table 2:** Characteristics of suicide cases in which individual interviews were conducted (*n* = 10).

Characteristics of suicide cases	Number of cases	
Sex		
Male	9	(90%)
Female	1	(10%)
Cause of death		
Hanging	9	(90%)
Other	1	(10%)
Years from university enrollment to suicide		
Undergraduate program	7	(70%)
1–2 years	1	(10%)
3 years	2	(20%)
4 years	4	(40%)
Graduate program	3	(30%)
1 year	1	(10%)
2 years	2	(20%)
History of using counseling services		
Yes	1	(10%)
No	9	(90%)
Living situation		
Living alone	8	(80%)
Living with family	2	(20%)
Discoverer/witness (overlap present)		
Student/friend	6	(60%)
Family	2	(20%)
Faculty members	2	(20%)
Unknown	1	(10%)
Cause of death communicated to related students (overlap present)		
Suicide	9	(90%)
Non‐suicide	2	(20%)

### Impact of exposure to suicide

Table 3 shows the characteristics of the 129 participants who were exposed to suicide among the 10 cases described above. Based on the IES‐R‐J cutoff score (24/25), students were divided into two groups. Tests of independence revealed significant associations between high IES‐R‐J scores and the following factors: sex (*χ* ^2^(1) = 7.90, *p* = 0.005, Cramer's *V* = 0.25), being in the same academic year as the deceased (*χ* ^2^(1) = 7.06, *p* = 0.008, Cramer's *V* = 0.23), and being the discoverer or witness (*p* < 0.001, Cramer's *V* = 0.38). No significant associations were found for being in the same faculty as the deceased (*χ* ^2^(1) = 3.14, *p* > 0.05, Cramer's *V* = 0.16) or for the reason for being selected for an individual interview (*p* > 0.05, Cramer's *V* = 0.17).

**Table 3:** Characteristics of interviewees and Japanese‐language version of the Impact of Event Scale‐Revised (IES‐R‐J) score.

Characteristics of interviewees	IES‐R‐J		*χ* ^2^ (df = 1)	*p*	Effect size Cramer's *V*
	<25 (*n* = 88)	≥25 (*n* = 41)			
Sex					
Male	78 (74%)	28 (26%)	7.90	0.005	0.25
Female	10 (43%)	13 (57%)			
Year of enrollment					
Same academic year	24 (53%)	21 (47%)	7.06	0.008	0.23
Different academic year	64 (76%)	20 (24%)			
Faculty affiliation					
Same faculty	67 (73%)	25 (27%)	3.14	NS	0.16
Different faculty	21 (57%)	16 (43%)			
The reason for being selected for an individual interview					
Same laboratory	61 (73%)	22 (27%)		NS^a^	0.17
Same club	25 (61%)	16 (39%)			
Other friends	2 (40%)	3 (60%)			
Discoverer or witness of suicide					
Discoverer/witness	3 (20%)	12 (80%)		<0.001^b^	0.38
Other	85 (75%)	29 (25%)			

^a^ Fisher–Freeman–Halton's exact test.

^b^ Fisher's exact test.

### Analysis of time‐related differences

**Figure 2:**
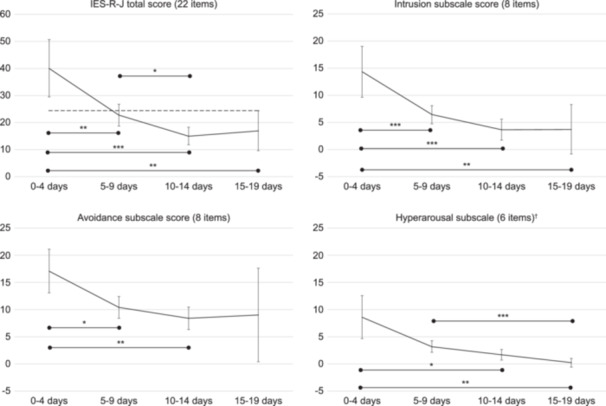
Multiple comparisons of changes in the Japanese‐language version of the Impact of Event Scale‐Revised (IES‐R‐J) scores. This figure shows the time‐related differences in the mean scores of the total scores (*n* = 127) and subscale scores (*n* = 76) of IES‐R‐J. Error bars represent the 95% confidence interval (CI). The broken line is the cutoff score (24/25) used in post‐traumatic stress disorder (PTSD) screening. Note: *p < 0.05; **p < 0.01; ***p < 0.001; ^†^Games–Howell test; error bars, 95% confidence interval.

Table 4 and Figure 2 show the time‐related trajectory of the IES‐R‐J scores at 5‐day intervals. A one‐way anova showed a significant main effect of time on total IES‐R‐J scores (*F*(3, 123) = 9.80, *p* < 0.001, *η* ^2^ = 0.19). Post hoc comparisons (Tukey's HSD) revealed that scores at 0–4 days were higher than those at 5–9 days (*p* = 0.003, Cohen's *d* = 1.14), 10–14 days (*p* < 0.001, Cohen's *d* = 2.14), and 15–19 days (*p* = 0.002, Cohen's *d* = 1.94), and scores at 5–9 days were higher than those at 10–14 days (*p* = 0.019, Cohen's *d* = 0.56). The mean total IES‐R score peaked immediately after the suicide, declined over time, and fell below the cutoff value by 5–9 days. Even the upper bound of the 95% confidence interval was below the cutoff by 10–14 days.

**Table 4:** Results of anova of the Japanese‐language version of the Impact of Event Scale‐Revised (IES‐R‐J) scores with time as a factor.

	*n*	*M*	SD	*F* (df)	*p*	*η* ^2^
**IES‐R‐J total (22 items, *n* = 127)**						
0–4 days	9	40.11	13.81	9.80 (3, 123)	<0.001	0.19
5–9 days	59	22.75	15.44			
10–14 days	50	15.04	11.37			
15–19 days	9	17.00	9.62			
**Subscale (*n* = 76)**						
Intrusion (8 items)						
0–4 days	9	14.33	6.08	10.00 (3, 72)	<0.001	0.29
5–9 days	40	6.45	5.25			
10–14 days	23	3.70	4.45			
15–19 days	4	3.75	2.87			
Avoidance (8 items)						
0–4 days	9	17.11	5.23	5.15 (3, 72)	0.003	0.18
5–9 days	40	10.43	6.30			
10–14 days	23	8.39	4.75			
15–19 days	4	9.00	5.42			
Hyperarousal (6 items)						
0–4 days	9	8.67	5.15	15.43^a^ (3, 23.70)	<0.001	0.32
5–9 days	40	3.20	3.29			
10–14 days	23	1.70	2.24			
15–19 days	4	0.25	0.50			

^a^ Welch's anova.

Subscale analyses (*n* = 76) also showed time effects: intrusion (*F*(3, 72) = 10.00, *p* < 0.001, *η* ^2^ = 0.29), avoidance (*F*(3,72) = 5.15, *p* = 0.003, *η* ^2^ = 0.18), and hyperarousal (Welch's *F*(3, 23.70) = 15.43, *p* < 0.001, *η* ^2^ = 0.32). The intrusion subscale scores at 0–4 days were higher than on 5–9 days (*p* < 0.001, Cohen's *d* = 1.46), 10–14 days (*p* < 0.001, Cohen's *d* = 2.16), and 15–19 days (*p* = 0.004, Cohen's *d* = 1.96). The avoidance subscale scores at 0–4 days were higher than at 5–9 days (*p* = 0.012, Cohen's *d* = 1.09) and 10–14 days (*p* = 0.001, Cohen's *d* = 1.79). For the hyperarousal subscale, differences were seen between 0–4 and 10–14 days (*p* = 0.015, Cohen's *d* = 2.12), 0–4 and 15–19 days (*p* = 0.05, Cohen's *d* = 1.91), and 5–9 and 15–19 days (*p* < 0.001, Cohen's *d* = 0.93). Symptoms for each subscale were highest immediately after the event and gradually decreased.

**Figure 3:**
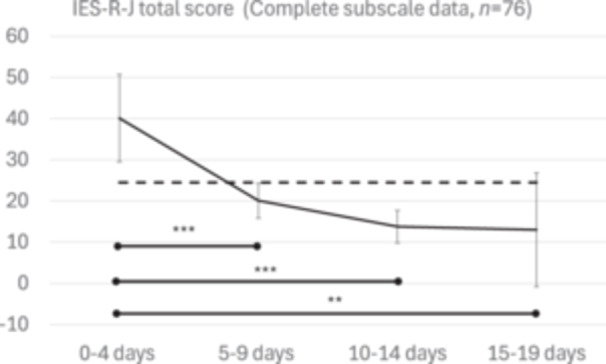
Sensitivity analysis. This figure shows the time‐related differences in the mean scores of total Japanese‐language version of the Impact of Event Scale‐Revised (IES‐R‐J) scores conducted within the complete‐case subset (*n* = 76). Error bars represent the 95% confidence interval (CI). The broken line is the cutoff score (24/25) used in post‐traumatic stress disorder (PTSD) screening. Note: *p < 0.05; **p < 0.01; ***p < 0.001; error bars, 95% confidence interval.

Table 5 presents the missing data analysis, comparing participants with complete subscale data (*n* = 76) to those with incomplete subscale data (*n* = 51). Participants with complete data were more likely to belong to the same faculty as the deceased, and their selection for individual interviews was more often attributed to being in the same laboratory (*p* < 0.001). However, no significant differences were found between participants with and without complete data in sex, year of enrollment, or status as a discoverer/witness of the suicide. Figure 3 shows the results of the sensitivity analysis including only participants with complete subscale scores (*n* = 76); scores were highest at 0–4 days and declined in subsequent periods (*F*(3, 72) = 10.87, *p* < 0.001, *η* ^2^ ＝ 0.31). Both the mean scores and the upper bound of the 95% confidence fell below the cutoff value by 5–9 days.

**Table 5:** Missing data analysis.

	Subscale data				
IES‐R‐J total	Complete (*n* = 76)	Missing (*n* = 51)	*t* (df = 125)	*p*	Effect sizeCohen's *d*
Mean	20.2	21.1	−0.34	NS	0.06

Characteristics of interviewees			*χ* ^2^ (df = 1)	*p*	Cramer's *V*
Sex					
Male	66 (63%)	38 (37%)	3.13	NS	0.16
Female	10 (43%)	13 (57%)			
Year of enrollment					
Same academic year	29 (64%)	16 (36%)	0.61	NS	0.07
Different academic year	47 (57%)	35 (43%)			
Faculty affiliation					
Same faculty	72 (80%)	18 (20%)	52.2	<0.001	0.64
Different faculty	4 (11%)	33 (89%)			
The reason for being selected for an individual interview					
Same laboratory	68 (82%)	15 (18%)		<0.001^a^	0.67
Same club	4 (10%)	35 (90%)			
Other friends	4 (80%)	1 (20%)			
Discoverer or witness of suicide					
Discoverer/witness	10 (67%)	5 (33%)		NS^b^	0.05
Other	66 (59%)	46 (41%)			

Abbreviation: IES‐R‐J, Japanese‐language version of the Impact of Event Scale‐Revised.

^a^ Fisher–Freeman–Halton's exact test.

^b^ Fisher's exact test.

### Analysis of factors affecting acute stress symptoms

Table 6 presents descriptions extracted from interview records of students' relationships with the deceased (*n* = 27) and their vulnerabilities (*n* = 12). Relationship descriptions included statements such as: “frequent opportunities to participate in shared activities within the same group (e.g., laboratory, club),” “having known each other for a long time,” “regular interactions due to physical proximity, such as adjacent seats in a lab, leading to frequent meetings and conversations,” and “the student regarding the deceased as a close friend.” Descriptions of personal vulnerabilities included: “a past loss of someone close, such as a friend, family member, or relative,” “a history of previous counseling, psychiatric care, or emotional instability,” “prior involvement in life‐threatening situations, either personal or involving others,” and “ongoing concerns related to life‐or‐death circumstances.”

**Table 6:** Description within the interview record.

	*n*
Descriptions related to the student's relationship with the deceased	
Frequent opportunities to participate in shared activities within the same group (e.g., laboratory, club).	10
Had known each other for a long time.	8
Regular interactions due to physical proximity, such as adjacent seats in a lab, led to frequent meetings and conversations.	5
The student regarded the deceased as a close friend.	4
Descriptions related to the postvention subject's vulnerability	
A past loss of someone close, such as a friend, family member, or relative.	5
A history of previous counseling, psychiatric care, or emotional instability.	4
Prior involvement in life‐threatening situations, either personal or involving others.	2
Ongoing concerns related to life‐or‐death circumstances.	1

Table 7 presents the results of the stepwise multiple regression analysis with the IES‐R‐J score as the dependent variable. Significant independent variables retained in the model included sex (*β* = 0.28, *p* < 0.001), being a discoverer or witness (*β* = 0.25, *p* = 0.001), the number of days from the suicide to the IES‐R‐J assessment (*β* = −0.21, *p* = 0.008), documented relationship with the deceased (*β* = 0.33, *p* = 0.001), and documented vulnerability (*β* = 0.18, *p* = 0.024). “Being in the same academic year” was excluded from the model. The model explained 39% of the variance (adjusted *R* ^2^ = 0.39) and yielded a multiple correlation coefficient of 0.65. anova also showed that the model was significant (*F*(5, 111) = 16.08, *p* < 0.001). The variance inflation factor (VIF) values ranged from 1.02 to 1.15, indicating no issues with multicollinearity.

**Table 7:** Factors affecting the Japanese‐language version of the Impact of Event Scale‐Revised (IES‐R‐J) scores.

Independent variables	*B*	SE	*β*	*p*	VIF
Sex (0: male, 1: female)	10.57	2.78	0.28	<0.001	1.02
Discoverer or witness of the suicide (0: no, 1: yes)	11.37	3.49	0.25	0.001	1.15
Number of days from the suicide to the IES‐R‐J assessment	−0.82	0.30	−0.21	0.008	1.10
Presence of a description of a relationship with the deceased student (0: no, 1: yes)	11.86	2.66	0.33	<0.001	1.06
Presence of vulnerability information in interview records (0: no, 1: yes)	8.79	3.84	0.18	0.024	1.14

*Note*: Adjusted *R* ^2^ = 0.39.

Abbreviations: *β*, standardized partial regression coefficient; *B*, partial regression coefficient; SE, standard error; VIF, variance inflation factor.

## DISCUSSION

This study identified several factors associated with elevated IES‐R‐J scores: female sex, being a discoverer or witness to suicide, shorter time since the suicide, having a close relationship with the deceased, and documented individual vulnerability. These findings are consistent with previous studies; in the present study, being a discoverer or witness corresponds to “degree of exposure,” a relationship with the deceased to “familiarity,” and documented individual vulnerability to “unique personal vulnerabilities” proposed by Brock et al.^16^

While previous studies have focused on the mid‐ to long‐term effects of exposure to suicide,^6^, ^10^, ^11^, ^12^ the present study focused on the immediate aftermath. Our results showed that acute stress symptoms among students exposed to suicide were highest immediately after the event and declined below the cutoff value within 5–14 days. As the IES‐R‐J data were collected only from the first assessment, these reductions likely reflect natural recovery rather than intervention effects. This suggests that although many students experience acute psychological distress immediately following exposure, a substantial number may recover without intervention.

Many participants (*n* = 51) had only total scores, with subscale scores missing. Missingness may reflect faculty‐level record‐keeping practices and was not completely random. However, no significant differences were observed between participants with complete and incomplete data regarding key psychological outcomes, and sensitivity analyses yielded comparable results. Accordingly, missing data influence appears limited.

The time‐related analysis of total IES‐R‐J scores in the complete‐case subset (*n* = 76) yielded results consistent with the full sample (*n* = 127), including similar direction/magnitude of differences and symptom decline trends. This indicates minimal bias from systematic differences between participants with complete and incomplete subscale data.

However, the potential for natural recovery does not negate intervention need. Exposure to suicide adversely affects mental state, academic performance, and daily functioning. Furthermore, acute stress reactions increase suicide risk^25^ and may trigger suicidal behaviors. If these reactions progress to PTSD, the risk of suicide/suicidal behavior escalates further.^26^, ^27^ Our results showed prioritized individual interviews for discoverers or witnesses or when suicide was widely known, supporting mental health focus in such situations. Despite frequent psychological debriefing use as a post‐intervention strategy, its efficacy remains controversial, with some studies reporting limited benefits^28^ and potential harm.^24^ Therefore, caution should be exercised when implementing trauma‐focused interventions.

Emotional debriefing may delay recovery in individuals with high baseline hyperarousal symptoms.^24^ Our data show hyperarousal symptoms may persist longer than other symptoms (no 0–4 days vs. 5–9 days difference) even in the absence of intervention, suggesting avoidance of emotional expression early post‐suicide.

Acute stress symptoms peak post‐suicide, making it difficult to identify those most affected and those in need of support. Early intervention balances both potential benefits and risks; and thus, postventions require careful implementation. The broad dissemination of suicide‐related information within a university risks excess exposure, whereas targeted interventions risk overlooking individuals in need.

It is reasonable to prioritize postvention recipients using factors linked to high IES‐R‐J scores (Table 7). A balanced and practical approach involves starting small, assessing individual responses through interviews, and expanding the scope as per needs. Students exhibiting severe or prolonged symptoms require counseling, faculty support, and medical referral.

Although not addressed here, bereaved families' wishes strongly influence the postvention decision‐making process.^15^ Moreover, media reporting affects suicidal behavior,^29^, ^30^ and the widespread use of social media amplifies and disseminates its psychological impact.^31^ Suicide prevention demands flexible, case‐tailored responses.

This study had several limitations. First, non‐standardized postvention procedures/documentation varied by practitioner. Although sensitivity analyses using the complete‐case subset suggested that missing subscale data did not substantially bias the time‐related findings, the potential impact of unobserved differences between complete and incomplete cases could not be fully examined. Information on students who did not request individual interviews was unavailable in the analyzed records, precluding assessment of selection bias. Second, the single‐university design limits generalizability. Shinshu University has a high proportion of students living away from their families (91.8%)^32^ compared to the national average for Japanese university students (40.9%),^33^ where friend discoverers predominate over family. Differences in institutional environments, cultural backgrounds, and student support systems may affect outcomes. Additionally, the generalizability of the findings toward Western and other multi‐cultural settings remains uncertain owing to Japan's unique norms surrounding suicide and postvention. Third, these IES‐R‐J scores represent cross‐sectional, not intra‐individual longitudinal changes. Therefore, individual differences in IES‐R‐J scores may have influenced the results. Furthermore, the specific period, location, and non‐random sampling also pose limitations, introducing biases into the data. Future research should include data from multiple institutions and develop standardized interview protocols and documentation formats. However, excessive standardization may lead to uniform interventions that are unsuitable for all cases. Postvention approaches should balance standardization and flexibility for individualized and context‐sensitive support.

## CONCLUSION

This study examined the factors influencing acute stress symptoms in university students exposed to suicide, using postvention records. Results showed sex, discoverer or witness to suicide, documented relationship with the deceased, and documented personal vulnerability were associated with the severity of acute stress symptoms. Symptoms subsided naturally over time. Effective postvention may prioritize support for individuals with multiple risk factors, using individual interviews to identify and expand support for others in need.

## AUTHOR CONTRIBUTIONS

*Conceptualization and methodology*: Isamu Yamazaki, Tohru Takahashi, and Hiroshi Morita. *Investigation*: Isamu Yamazaki. *Formal analysis*: Isamu Yamazaki and Daimei Sasayama. *Writing—original draft*: Isamu Yamazaki and Tohru Takahashi. *Supervision*: Daimei Sasayama, Hiroshi Morita, and Shinsuke Washizuka. *Writing—review and editing*: All authors. All authors have read and agreed to the published version of the manuscript.

## CONFLICT OF INTEREST STATEMENT

The authors declare no conflicts of interest.

## ETHICS APPROVAL STATEMENT

This study was approved by the Biological and Medical Research Ethics Committee of Shinshu University School of Medicine (Approval No. 6144).

## PATIENT CONSENT STATEMENT

The requirement for patient consent was waived by the ethics committee because the study used only non‐identifiable retrospective data and employed an opt‐out procedure.

## CLINICAL TRIAL REGISTRATION

N/A.

## Data Availability

The data that support the findings of this study are available on request from the corresponding author. The data are not publicly available due to privacy or ethical restrictions.
